# Morphological and Radiological Study of Ossified Superior Transverse Scapular Ligament as Potential Risk Factor of Suprascapular Nerve Entrapment

**DOI:** 10.1155/2014/613601

**Published:** 2014-04-03

**Authors:** Michał Polguj, Marcin Sibiński, Andrzej Grzegorzewski, Michał Waszczykowski, Agata Majos, Mirosław Topol

**Affiliations:** ^1^Department of Angiology, Medical University of Łódź, Ulica Narutowicza 60, 90-136 Łódź, Poland; ^2^Clinic of Orthopaedics and Paediatric Orthopaedic, Medical University of Łódź, Ulica Drewnowska 75, 91-002 Łódź, Poland; ^3^Clinic of Arthroscopy, Minimally Invasive Surgery and Sports Traumatology, Medical University of Łódź, Ulica Żeromskiego 113, 90-549 Łódź, Poland; ^4^Department of Radiological and Isotopic Diagnosis and Therapy, Medical University of Łódź, Ulica Żeromskiego 113, 90-549 Łódź, Poland; ^5^Department of Normal and Clinical Anatomy, Medical University of Łódź, Ulica Narutowicza 60, 90-136 Łódź, Poland

## Abstract

The suprascapular notch is covered superiorly by the superior transverse scapular ligament. This region is the most common place of suprascapular nerve entrapment formation. The study was performed on 812 specimens: 86 dry scapulae, 104 formalin-fixed cadaveric shoulders, and 622 computer topography scans of scapulae. In the cases with completely ossified superior transverse scapular ligament, the following measurements were performed: proximal and distal width of the bony bridge, middle transverse and vertical diameter of the suprascapular foramen, and area of the suprascapular foramen. An ossified superior transverse scapular ligament was observed more often in men and in the right scapula. The mean age of the subjects with a completely ossified superior transverse scapular ligament was found to be similar than in those without ossification. The ossified band-shaped type of superior transverse scapular ligament was more common than the fan-shaped type and reduced the space below the ligament to a significantly greater degree. The ossified band-shaped type should be taken into consideration as a potential risk factor in the formation of suprascapular nerve entrapment. It could explain the comparable frequency of neuropathy in various populations throughout the world despite the significant differences between them in occurrence of ossified superior transverse scapular ligament.

## 1. Introduction

The superior transverse scapular ligament (STSL) is a fibrous band connecting two borders of suprascapular notch (SNN) on the upper border of the scapula. The suprascapular nerve (SN) passes below the ligament through the opening [1]. This area can result in compression or injury to the suprascapular nerve, which can result in suprascapular nerve entrapment syndrome, first described by 1936 by André Thomas [2]. The basic clinical manifestations of this neuropathy include deep, dull and diffuse pain of the posterior and lateral aspects of the shoulder, weakened abduction and rotation of the upper extremity in the glenohumeral joint, and progressive atrophy of the supra- and infraspinatus muscles [1, 3].

One of the most important risk factors of suprascapular nerve entrapment is a completely ossified superior transverse scapular ligament [4–7]. Recent histological and immunohistochemical studies have revealed signs of neural degeneration in all nerve specimens with STSL ossification [7]. On the other hand, in some populations, the frequency of ossified STSL is estimated to be 30.56% and is not correlated with an increase of cases of suprascapular nerve entrapment [8]. Probably this may depend on the type of ossified STSL. However, in the etiopathology of suprascapular nerve entrapment, less attention has been devoted to the influence of the shape of the bony bridge created by an ossified STSL. More research in this area would undoubtedly prove to be of great clinical value, especially for the orthopaedist.

This article reports the largest study so far on the morphology and variations of ossified STSL. It also uses three independent methods to measure the space below the STSL which is available for the passage of the SN. This study is unique, and so far all earlier observations of the ossified STSL have been only macroscopic in nature and not supported by any calculations.

## 2. Materials and Methods

**Figure 1:**
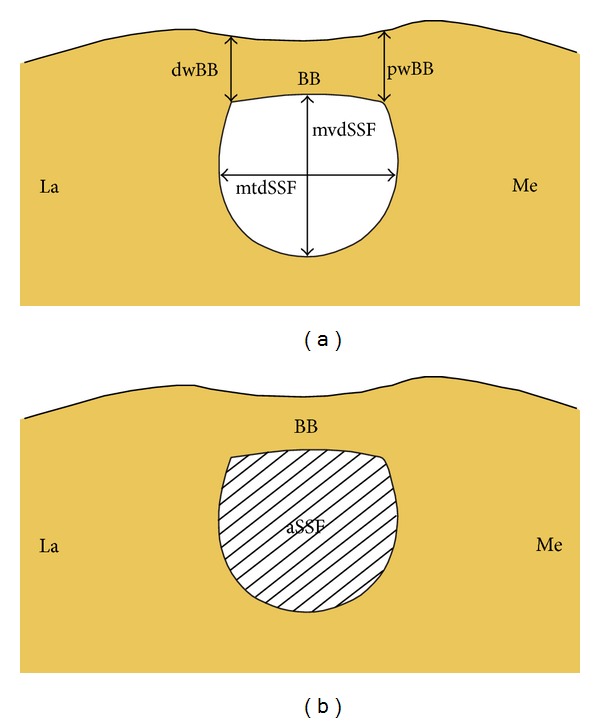
Schematic representation of the arrangements of the structures in the suprascapular region of the scapula with completely ossified superior transverse scapular ligament: aSSF: area of the suprascapular foramen, BB: bony bridge, dwBB: distal width of the bony bridge, pwBB: proximal width of the bony bridge, mtdSSF: middle transverse diameter of the suprascapular foramen, mvdSSF: middle vertical diameter of the suprascapular foramen, La: lateral, and Me: medial.

The study was performed on 812 specimens. It consists of three independent parts: an anatomical study of dry scapulae, an anatomical study of formalin-fixed cadaveric shoulders, and a radiological study of computer tomography scans of scapulae. The research project and all procedures were approved by the Bioethics Commission of our Medical University. All donors gave informed consent to dispose their bodies for scientific purposes. The suprascapular regions of all 812 scapulae were analysed. In the specimens with a completely ossified STSL, the following measurements were collected (Figure 1):

1. proximal width of the bony bridge (pwBB)—the maximal distance between the superior and inferior borders of the bony bridge at its proximal end (Figure 1(a));
2. distal width of the bony bridge (dwBB)—the maximal distance between the superior and inferior borders of the bony bridge at its distal end (Figure 1(a));
3. area of the suprascapular foramen (aSSF)—the area limited by the inferior border of the ossified STSL and osseous borders of the SSN (Figure 1(b));
4. the middle vertical diameter of the suprascapular foramen (mvdSSF)—the maximum value of the longitudinal measurements taken in the vertical plane from a point half way along the length of the bony bridge and the deepest point of the suprascapular notch (Figure 1(a));
5. the middle transverse diameter of the suprascapular foramen (mtdSSF)—the maximal distance between the proximal and distal margins of the SSN, taken in the horizontal plane in half dimension of MVD, perpendicular to it (Figure 1(a)).

**Figure 2:**
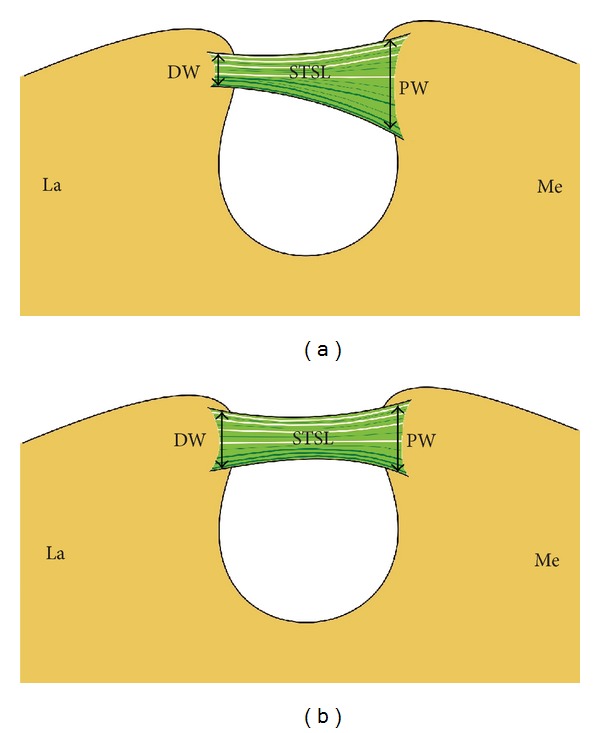
Schematic arrangements of types of superior transverse scapular ligament (STSL): (a) fan-shape type of STSL and (b) band-shape type of STSL. DW: distal width, PW: proximal width, STSL: superior transverse scapular ligament, La: lateral, and Me: medial.

Based on the newest description of STSL variations [9], the bony bridge was classified as either fan-shaped or band-shaped. A fan-shaped type is defined as having a proximal width (PW) at least twice as wide as the distal width (PW/DW >2) (Figure 2(a)) while a band-shaped type is defined as the opposite, the ratio between PW and the DW being less than 2 (DW/PW <2) (Figure 2(b)).

### 2.1. Anatomical Study on Dry Scapulae

A total of 86 dried human dry scapulae were included in the study: 40 left and 46 right. The age and sex of the donors were unknown. The exclusion criterion was the presence of injuries which made measurement collection impossible.

### 2.2. Anatomical Study on Formalin-Fixed Cadaveric Shoulders

The anterior aspect of 104 embalmed adult human shoulders was dissected: 46 women and 58 men. The age and sex of the donors were known. In all cadaveric shoulders, the skin was separated from the deltoid, trapezius, and pectoralis major after horizontal incision along the clavicle. Next, the trapezius and the deltoid muscles were removed. After retraction of the supraspinatus muscle, the superior border of the scapula was visualized. Next, the suprascapular notch region was analyzed.

All anatomical investigations were performed in the Chair of Anatomy, Medical University of Łódź. In both anatomical studies, to normalize measurements, all photographic documentation was obtained from a standardized position of the camera and shoulder. All scapulae and shoulders were fixed with an adjustable clamp and ring stand with the same distance from the camera. The same scale was used for all measurements. Digital photographic documentation was processed with use of MultiScanBase v.18.03 software (Computer Scanning System II, Warsaw, Poland), which allows images to be analysed with a particular emphasis on the measurement functions. The usefulness and correctness of this method were confirmed with the Bland-Altman plot and R2 value described in a previous study [10].

### 2.3. Radiological Study

The study was performed on a retrospective analysis of 622 computer tomography scans of shoulders taken as part of a standard CT chest protocol. Dual-phase helical CT was performed with a 32-row MDCT scanner (Toshiba Aquilion 32; Toshiba Medical System, Japan) for pathology of the lungs or cardiovascular system. The criteria of exclusion were any scapular pathology or metastases to bone. The values of the scapula were measured using Vitrea 2 system software (Vital Images, Plymouth, MN, US).

### 2.4. Statistical Analysis

Statistical analysis for all parameters was performed for use Statistica 10 software (StatSoft Polska, Cracow, Poland). The normality of data distribution was checked by means of the Shapiro-Wilk test and variance equality by Levene's test. Comparison of the area of the suprascapular opening (aSSF) between samples with fan-shaped and band-shaped ossified STSLs was performed with the Student *t*-test for independent variables. The *χ* ^2^ test was used to assess the statistical differences regarding the frequency of occurrence of ossified STSL between both sexes. For each used statistical test a *P* level of < 0.05 was accepted as statistically significant.

## 3. Results

For all groups, an ossified superior transverse suprascapular ligament was identified in 5.42% of cases (44/812). It was observed more often in men (6.4%—26/406) than in women (3.75%—12/320). This difference between both sexes was statistically significant according to the *χ* ^2^ test (*P* = 0.01537). The mean age of the subjects with a completely ossified superior transverse scapular ligament (61.9 years) was found to be similar than in those without (62.13 years). An ossified STSL was observed more often on the right side—52.3% (23/44) than left side—47.7% (32/44).

### 3.1. Anatomical Study on Dry Scapulae

**Figure 3:**
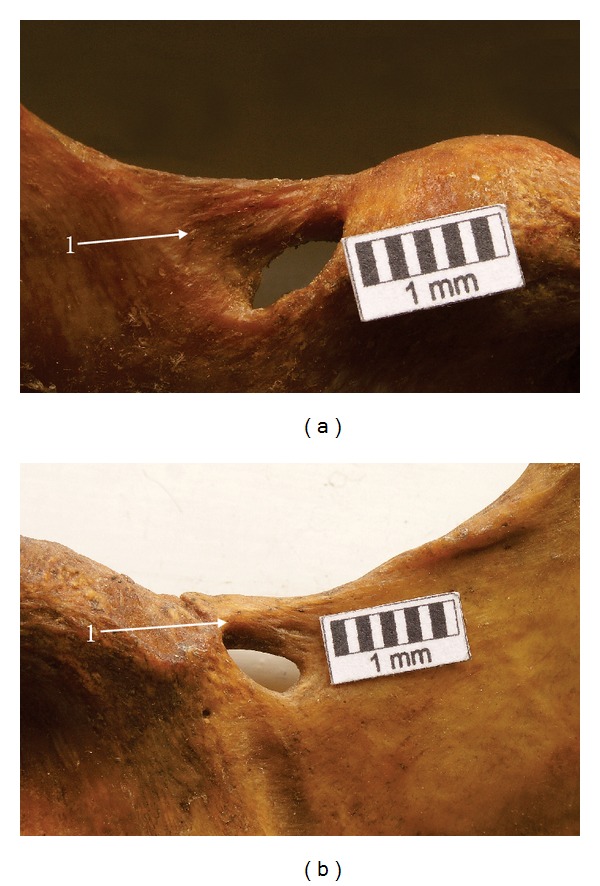
Suprascapular region of the dry scapula. 1: superior transverse scapular ligament (STSL). (a) Fan-shape type of ossified STSL, (b) band-shape type of ossified STSL.

Based on the measurements of the maximal proximal and distal widths of the bony bridge and by applying metric criteria used in classification of the STSL types [10], it was found that the band-shaped type (4.7%—4/86) (Figure 3(b)) was more often ossified than the fan-shaped type (2.3%—2/86) (Figure 3(a)). Also the mean surface area of the foramen formed in this way was greater in the fan-shaped than in the band-shaped type (50.75 mm^2^ and 30.43 mm^2^, resp.). However, the number of specimens was too low to perform any statistical analysis. The mean middle vertical diameter and mean transverse diameter of the suprascapular foramen were greater in specimens with the fan-shaped (7.15 mm and 8.75 mm, resp.) than in the band-shaped STSL (7.03 mm and 5.35 mm, resp.).

### 3.2. Anatomical Study on Formalin-Fixed Cadaveric Shoulders

**Figure 4:**
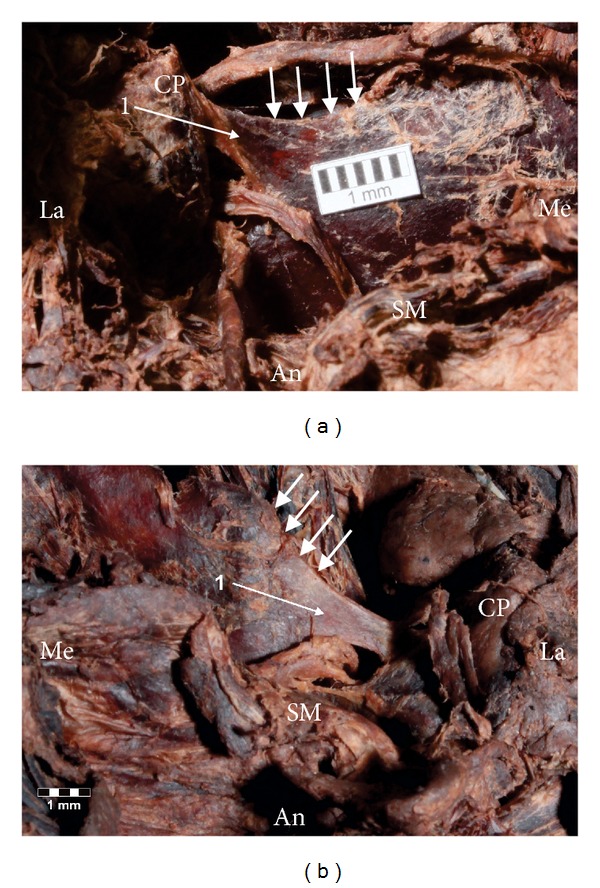
Formalin-fixed cadaveric shoulders of suprascapular region. 1: superior transverse scapular ligament (STSL). (a) Fan-shaped type of ossified STSL, (b) band-shaped type of ossified STSL. The arrows indicate superior border of the scapula. An: anterior; CP: coracoid process; La: lateral; Me: medial; SM: subscapular muscle.

Taking into consideration the metric criteria used in the classification of the STSL types, the band-shaped type was found to be ossified more often (3.9%—4/104) (Figure 4(b)) than the fan-shaped type (2.9%—3/104) (Figure 4(a)). The surface area of the suprascapular foramen was greater in the fan-shaped type (59.62 mm^2^) than in the band-shaped type (40.59 mm^2^). However, the number of specimens was too low to perform any statistical analysis. Also, the mean middle vertical diameter and mean transverse diameter of the suprascapular foramen were greater in specimens with the fan-shaped STSL (8.86 mm and 9.13 mm, resp.) than in the band-shaped STSL (6.63 mm and 6.29 mm, resp.).

### 3.3. Radiological Study

**Figure 5:**
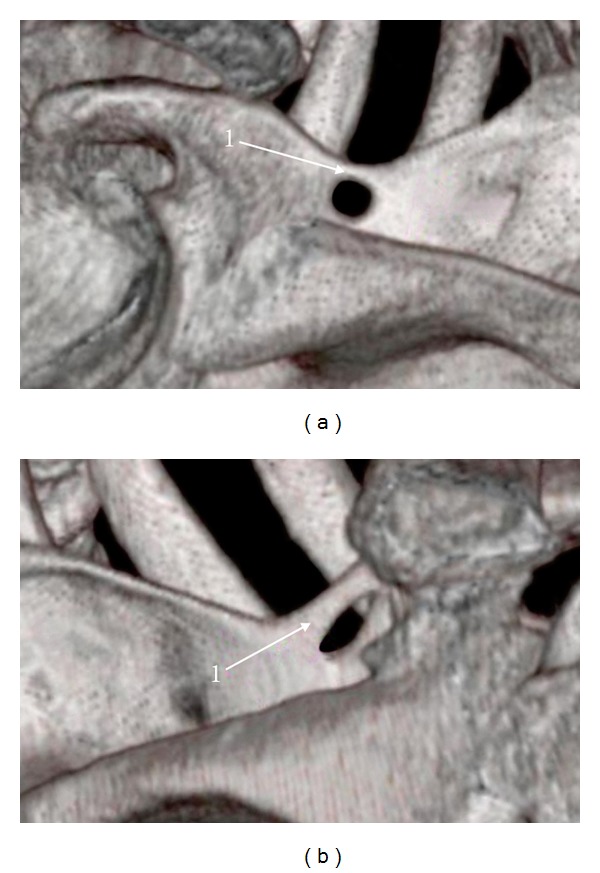
Three-dimensional volume rendering (VR) multidetector computed tomography (MDCT) of the scapula: 1: superior transverse scapular ligament (STSL). (a) Fan-shaped type of ossified STSL, (b) band-shaped type of ossified STSL.

Among the analyzed scapulae in CT, a completely ossified superior transverse suprascapular ligament was found on the left side in 15 patients and on the right side in 16 patients. The band-shaped type (Figure 5(b)) was more often ossified than the fan-shaped type (Figure 5(a)) (4.02%—25/622 and 0.96%—6/622, resp.). The mean surface area of the suprascapular foramen was higher in the fan-shaped type (49.11 mm^2^) than in the band-shaped type (34.37 mm^2^). According to the Student's* t*-test, the mean surface areas of the suprascapular foramen in the ossified fan-shaped and band-shaped types were significantly different (*P* = 0.01394). The mean middle vertical diameter and mean transverse diameter of the suprascapular foramen were greater in specimens with the fan-shaped STSL (7.48 mm and 8.66 mm, resp.) than in the band-shaped STSL (7.1 mm and 5.66 mm, resp.).

## 4. Discussion

According to Tubbs et al., the most important predisposing factor for suprascapular nerve entrapment is a completely ossified superior transverse suprascapular ligament. In their analysis, all specimens with ossified STSL displayed signs of neural degeneration in the suprascapular nerve [7]. Also, Ticker et al. [5] and Rengachary et al. [4] note that an ossified STSL can be a risky factor for surgical explorations during a suprascapular nerve decompression.

The frequency of completely ossified superior transverse scapular ligament varies throughout the world. In the European population, its occurrence is estimated to range from 1.5% to 12.5% of cases: 1.5% in Finland [11], 3.6–6.1% in Italy [12, 13], 4.72% in Poland [14], 5–6.5% in France [15, 16], 7.3% in Germany [17], and 6.0–12.5% in Turkey [18, 19]. Complete ossification of the STSL in the US population was found in 3.7–6.34% of cases [4–6, 10, 20–22]. Several studies have also been performed on other populations from another continents: Africa, Kenya, with 3% of cases [23] and Egypt with 13.6% of cases [24], Asia, China with 4.08% of cases [25], and South America, Brazil with 30.76% [8]. However, great variability of ossified STSL is shown worldwide (Table 1). In some populations, it was very rare, for example, Alaskan Eskimos, 0.3% [26], or Native Americans, 2.1–2.9% [24], but, in others, it was much more frequent, for example, Brazilian, 30.76% [8]. Explanation of such diversity is not known. We suggest that the occurrence of the bony bridge formed by ossified STSL could have a genetic basis. It is supported by Cohen et al. study. They describe a familiar case of calcification of the STSL affecting a 58-year-old man and his son; both incidences are being associated with suprascapular nerve entrapment and attendant clinical symptoms of pain, weakness, and atrophy of the supraspinatus muscle [27].

Our results are similar to those observed by Silva et al., who observed that 36/68 of shoulders (52.94%) were ossified on the right side and 32/68 (47.05%) on the left with our results being 52.3% on the right and 47.7% on the left [8]. According to Tubbs et al., the presence of foramen scapulae was significantly greater on the right side and in male cadavers [7]. However, Albino et al. report that the characteristics of the patient, such as gender, age, and scapular dimensions, are not related to the dimensions and type of the suprascapular notch [12].

According to Polguj et al., nonossified the fan-shaped STSL (54.6%) is more common than the band-shaped type (41.9%) [9]. However, in our study, the band-shaped type of STSL was found to be ossified three times more frequently (33/44) than the fan-shaped type (11/44). It is possible that when the diameter of the suprascapular foramen is significantly reduced, the suprascapular artery passing below the STSL might exert blood pressure on the ligament, causing microtrauma finally resulting in ossification. This theory is supported by the findings of Moriggl et al. [28]. They described a small bony nodule in the central part of the STSL, comparable to a centre of ossification of a specimen which also demonstrated a lateral bony spur. Also, Tubbs et al. suggested that when the artery neighbours the nerve directly, it might exert blood pressure on the more fragile nerve, causing microtrauma to the nerve resulting in neuropathy [6].

Our study is the first one to distinguish variable morphology of ossified STSL. All recent observations are not supported by any statistical calculations and have only been performed at a macroscopic level; this study is the first to take a quantitative approach. According to our results, the mean area of the suprascapular foramen in the specimens with an ossified band-shaped STSL is significantly smaller than in those with a fan-shaped STSL. Therefore, we can confirm that band-shaped type of ossified STSL forms less space for the passage of the suprascapular nerve. Such an observation may explain why there is no increase in suprascapular nerve entrapment, even when the frequency of ossified STSL is very high, for example, 30.76% in the Brazilian population [8].

Gosk et al. state that peripheral nerves are highly susceptible to injury from stretching and compression. Both of these mechanisms result in nerve ischemia, edema, microenvironmental changes, and conduction impairment [1]. Also, according to Ringel et al. vascular microtrauma has also been postulated to cause nerve dysfunction [29]. In 1979, Rengachary et al. first proposed an etiopathogenesis of suprascapular nerve entrapment known as the* sling effect*. It assumes that during arm motion, the suprascapular nerve makes only minimal transitional movements. However, during maximal rotation, an angulated nerve can be pressed against the sharp bony margin of the suprascapular notch. Such repeated kinking irritates the nerve and induces microtrauma that can result in suprascapular neuropathy [4].

However, in our opinion, the etiopathogenesis of suprascapular nerve entrapment is more complex, and it depends on several factors. Presumably, the risk of this neuropathy can be increased by morphological variations in suprascapular region such as the presence of a bifid STSL [30, 31] or trifid STSL [32], a narrow V-shape of the suprascapular notch [4], a hypertrophied subscapular muscle [31], spinoglenoid ligament [33], or the presence of double suprascapular foramen [24].

According to a current bibliography search, males are approximately three to four times more likely to suffer from this neuropathy than females [3, 15]. We suppose that one of the anatomical factors which might explain this higher frequency of suprascapular neuropathy in males may be the significantly higher frequency of scapulae with a completely ossified STSL. What is more, although suprascapular nerve entrapment is uncommon, it would seem to be a demographic problem, because it mainly occurs in patients under 38 years of age [1, 3]. Nonspecific symptoms are often diagnosed late, when the supra- and infraspinatus muscles have atrophied. According to Gosk et al., the outcome of surgery depends on the length of time between first symptom onset and the treatment itself [1]. Also, Olivier [15] states that early decompression of the nerve at suprascapular notch has proven to be efficient procedure that restores shoulder function [34]. Sergides et al. conclude that arthroscopic decompression of the entrapped suprascapular nerve is technically challenging, but less invasive and potentially a more effective way to treat suprascapular neuropathy. It may also provide a more rapid recovery, especially in the rare case that the nerve is depressed by an ossified superior transverse scapular ligament [35]. Therefore, all morphological studies on variations of structures at the suprascapular region are potentially helpful in the diagnosis and treatment of this pathology.

## 5. Conclusion 

The ossified band-shaped superior transverse scapular ligament should be considered as potential risk factor in suprascapular nerve entrapment because the space below the bony bridge is significantly reduced compared with the case of the ossified fan-shaped ligament. It could explain the comparable frequency of neuropathy in various populations throughout the world despite the significant differences between them in occurrence of ossified superior transverse scapular ligament.

## Figures and Tables

**Table 1:** Frequency of ossifications of the superior transverse scapular ligament in different populations.

Researcher	Country	Ossification (%)	Number of studied specimens (*N*)
Kajava [11]	Finland	1.5	*N* = 133
Olivier [15]	France	5.0	*N* = 100
Vallois [13, 16]		6.5	*N* = 200
	Italy	6.1	*N* = 152
Albino et al. [12]		3.6	*N* = 500
Natsis et al. [17]	Germany	7.3	*N* = 423
Polguj et al. [14]	Poland	4.72	*N* = 616
Urgüden et al. [19]	Turkey	6.0	*N* = 100
Bayramoglu et al. [18]		12.5	*N* = 32
Edelson [22]	USA	3.7	*N* = 1000
Tubbs et al. [6]		3.7	*N* = 120
Rengachary et al. [4]		4.0	*N* = 211
Dunkelgrun et al. [21]		5.0	*N* = 623
Ticker et al. [5]		5.0	*N* = 79
Gray [10]		6.34	*N* = 1151
Sinkeet et al. [23]	Kenya	3.0	*N* = 138
Wang et al. [25]	China	4.08	*N* = 295
Hrdicka [24]	Egypt	13.6	*N* = 511
Silva et al. [8]	Brazil	30.76	*N* = 221
